# Ligament/Tendon Culture under Hypoxic Conditions: A Systematic Review

**DOI:** 10.34172/apb.2021.069

**Published:** 2020-10-20

**Authors:** Sholahuddin Rhatomy, Dwikora Novembri Utomo, Cita Rosita Sigit Prakoeswa, Fedik Abdul Rantam, Heri Suroto, Ferdiansyah Mahyudin

**Affiliations:** ^1^Doctoral Program, Faculty of Medicine, Universitas Airlangga, Surabaya, Indonesia.; ^2^Department of Orthopaedic and Traumatology, Dr. Soetomo General Hospital, Faculty of Medicine, Universitas Airlangga, Surabaya, Indonesia.; ^3^Department of Dermatology and Venereology, Dr. Soetomo General Hospital, Faculty of Medicine, Universitas Airlangga, Surabaya, Indonesia.; ^4^Virology and Immunology Laboratory, Microbiology Department, Faculty of Veterinary Medicine, Universitas Airlangga, Surabaya Indonesia.; ^5^Stem Cell Research and Development Center, Universitas Airlangga, Surabaya Indonesia.

**Keywords:** Hypoxic condition, Ligament, Tendon, Culture

## Abstract

The hypoxic environment is a substantial factor in maintenance, proliferation, and differentiation of the cell cultures. Low oxygen is known as a potent chondrogenesis stimulus in stem cells that is important for clinical application and engineering of functional cartilage. Hypoxia can potentially induce angiogenesis process by secretion of cytokines. This systematic review goal is to discover the effect of hypoxic condition on tendon/ ligament culture and the best oxygen level of hypoxia for in vitro and in vivo studies. We included 21 articles. A comprehensive review of this database confirms that the hypoxic condition is a substantial factor in the maintenance, proliferation, and differentiation of ligament/tendon cultures. Cell proliferation in the severe hypoxic (oxygen concentration of 1%) group at 24 h postcultivation was considered significant, but cell proliferation was markedly inhibited in the severe hypoxic group after 48 h.

## Introduction

Hypoxia is a physiological condition in the environment of various stem cells. In a previous study, the oxygen level for most cell cultures was maintained at approximately 20% in vitro.^1^ The native microenvironment in the body has a low oxygen pressure; on average, this pressure is approximately 12% in arterial blood and 3% in tissues, which may vary depending on the cell location.^1^

Hypoxia has been used in tissue cultivation. Some studies have reported a significant increase in the proliferation of cells cultured under low oxygen pressure.^2,3^ Comparisons of growth in normal and low oxygen levels during preconditioning showed that embryonic stem cells grew more efficiently with low oxygen. Among the spectrum of adult stem cells, Grayson et al. examined human bone marrow-derived mesenchymal stem cells (MSCs) and showed that low oxygen levels resulted in a 30-fold increase in cell growth compared to normoxic conditions.^1^

Tendon or ligament tears are frequent injuries that may disturb the patient’s quality of life. The healing process is often inadequate, leading to scar tissue formation. Various treatments for these injuries have been developed, including physiotherapy, pain medications, steroid injections, and surgical procedures. However, these therapeutic methods have not been satisfactory for patients. Stem cells have become a new therapeutic modality because of their ability to induce cell rejuvenation, stemness potential, and immunomodulatory activity. A systematic review by Ahmad et al. revealed that stem cell treatment has been applied to heal injured tendons in animals and humans. Among the various sources of stem cells, MSCs are highly useful for healing tendons and ligaments and preventing scar formation.^4,5^

Some studies have examined various levels of hypoxic conditions and their effects.^6^ However, limited evidence is available regarding the use of hypoxic conditions for ligament/tendon cultures. An in-depth review of in vitro and in vivo studies is needed to evaluate the effectiveness of hypoxic conditions for ligament/tendon cultures and to translate their use into clinical applications. Thus, this review aimed to determine the effect of hypoxic conditions and to identify the optimal oxygen level for use in in vivo and in vitro tendon/ligament cultures.

## Methods

### Review protocol

Our review aimed to elucidate the effect of hypoxic levels on tendon/ligament cultures as well as the optimal hypoxic condition (oxygen concentration) for in vitro and in vivo studies.

### Literature search and study selection

In May 2020, a database search was carried out in the Scopus, PubMed (Medline), Web of Science, Science Direct, Google Scholar, and Cochrane Library databases to identify all studies published in English that described the outcomes of ligament/tendon cultures under hypoxic conditions. A comprehensive search of the literature was performed based on the Animal Research: Reporting of In VivoExperiments (ARRIVE) guidelines and the Checklist for Reporting In vitro Studies (CRIS Guidelines).

The search keywords included “ligament”, “tendon”, “culture”, “condition”, “precondition”, and “hypoxic” alone and combined the Boolean operators “AND” or “OR”.

Two authors (DNU and SR) independently scanned for eligibility based on the title and abstract after duplicate and review articles were removed from the collected articles. These two authors also performed an additional search based on references to the included articles. The final search result was included in this review. The included articles were read by the authors to match the inclusion and exclusion criteria. Any discordance between authors was resolved through discussion.

### Outcome measures

The following outcome measures were analysed: 1) the most common hypoxic condition (oxygen concentration) used and 2) the main outcomes.

### Eligibility criteria

The following inclusion criteria were used: 1) peer-reviewed level 1 to 4 studies, 2) studies published in English, 3) studies of ligament or tendon cultures, 4) a description of the hypoxic condition (oxygen concentration), and 5) an obvious description of the outcomes. Studies that were not published in English, duplicate articles, review studies, and irrelevant articles were excluded.

### Assessment of methodological quality and risk of bias

The methodological quality of the included studies was assessed using ARRIVE and CRIS guidelines.^7,8^ Internal validity was assessed using the Systematic Review Center for Laboratory Animal Experimentation Risk of Bias tool (SYRCLE RoB tool).^9^ Two authors (DNU and SR) independently performed all assessments.

### Data extraction and synthesis

Two authors (DNU and SR) independently analysed and recorded the data from the included studies. The following data were extracted: study design, types of animals used for in vivostudy, cell types for in vitrostudy, process for establishing animal or cell models, hypoxic condition, stem cell isolation procedures, interventions, comparisons, follow-up duration, primary outcomes of in vivoand in vitrostudies and their results, any significant deviation from the control, and other outcomes.

We also recorded the blinding method of data collection of the included studies when available. We collectively reported the types of animals used in the study, interventions, and duration of follow-up. We reported any adverse reactions, quantitative outcome measurements, and biomechanical tests for in vivostudies. For in vitrostudies, we reported cell proliferation, viability and migration; tenogenic differentiation; and immunomodulatory properties. We could not perform a meta-analysis because of the substantial heterogeneity of the data (i.e., different intervention levels of hypoxic conditions and different measurements of the outcomes).

## Results

### Study selection

**Figure 1:**
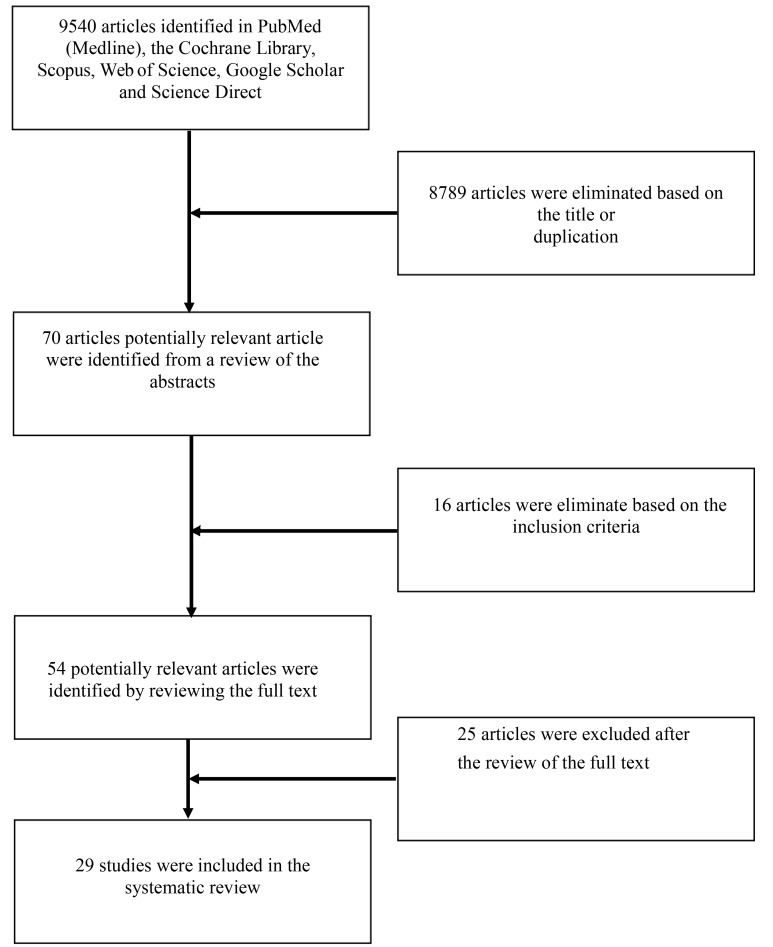
Flow chart of study process selection.

In total, 9540 articles were collected from the databases; 8789 articles were excluded based on the title or duplication. Title and abstract screening was conducted for 70 articles. Sixteen articles were excluded because they did not match the inclusion criteria. After enrolling 54 articles for full-text assessment, 25 were excluded due to insufficient description of hypoxic conditions (n = 21), short communications/reviews (n = 2), and multiple reports of the same experiment (n = 2). Ultimately, 29 articles were enrolled in this systematic review. The article selection process is provided in Figure 1.

### Characteristics of selected studies

Of the 29 studies, 24 (82.7%) were published between 2010 and April 2020. Human periodontal ligament-derived stem cells (86%) were the main cell type used among these studies. All included studies were conducted in vitro, but 2 studies also reported the results of an in vivo experiment (Table S1).^6,10^

### Stem cell source

Of the 29 studies, 27 (93%) used human stem cells, and only 2 studies used stem cells from an animal (rat). Moreover, 21 studies (72%) used the human periodontal ligament, whereas the remaining studies used the Achilles tendon or periodontal ligament from rats, human hamstring, or human anterior cruciate ligament (Table S1).

### Hypoxic conditions (oxygen concentration)

Fourteen studies performed a comparison with normoxic conditions (20% or 21% oxygen), three used 1% oxygen,^11-13^ five used 2% oxygen,^14-18^ two used 3% oxygen,^10,19^ and four studies compared various hypoxia levels, including 1%, 2%, and 5% oxygen;^6^ 1% oxygen and a reoxygenation group (from 1% to 20% oxygen);^14,20^ and 5% and 1% oxygen.^21^ Overall, 15 studies evaluated the effects of certain oxygen concentrations: 3 used 5% oxygen,^22-24^ 1 used 3% oxygen,^25^ 5 used 2% oxygen,^6,26-29^ 2 used 1% oxygen,^11,30^ 3 used less than 1% oxygen,^31-33^ and another used less than 0.1% oxygen^34^ (Table S1).

### Main outcomes

Seven studies (24%) reported increased cell proliferation.^6,14,16,23,30,35,36^ Four studies showed that hypoxic conditions increased alkaline phosphatase activity.^6,14,23,26^ Six studies revealed a significant increase in vascular endothelial growth factor (VEGF) expression.^14,20,24,26,31,34^ Eight studies reported a substantial increase in the expression of cytokines, such as interleukin (IL)-1β,^20,31,37^ IL-6,^11,20,31,37^ IL-8,^31^ IL-10,^10^ IL-37^10^ and tumour necrosis factor-α (TNF-α),^37^ in cells cultured under hypoxic conditions. Ito et al. observed no effects by hypoxia on the secretion of IL-1β, IL-6, IL-8, IL-17A, macrophage migration inhibitory factor, monocyte chemoattractant protein-1, TNF-α, and macrophage colony-stimulating factor in the culture media.^34^ Moreover, other studies revealed a significant increase in collagen type I^13^ and III^11,13^ and glycosaminoglycan (GAG).^13^

Six studies reported an increase in osteogenic differentiation.^15,17,18,26,30,35^ Hypoxic conditions also increased osteoclastogenesis,^29,32^ improved the maintenance of pluripotent stem cells,^16^ increased the expression of several mediators of apoptosis, and promoted tenocyte apoptosis^11^ (Table S1).

## Discussion

This study was a systematic review of the available literature describing ligament/tendon cultures under hypoxic conditions. In this review, we assessed the characteristics of the selected studies, stem cell sources, hypoxic conditions (i.e., oxygen concentration), and the main study outcomes.

The oxygen level of the tissue culture in the in vitro study was maintained at approximately 20%. Hypoxia plays an important role in marker secretion and especially in haematologic features, such as gene expression in haematopoietic stem cells (HSCs) and hypoxia-inducible factor-dependent expression in supporting cells. This suggests that hypoxia may improve HSC growth and maintenance during ex vivo culture.^38^ Hypoxia promotes MSC cultivation by improving cell expansion, osteogenesis increment, and bone and cartilage cell proliferation. Furthermore, MSC preculture under hypoxic conditions improved their regenerative potential.^1^

Matsuda et al. first studied ligament/tendon cultures using human periodontal ligament exposed to 5% oxygen,^23^ after which research on ligament or tendon cultures under hypoxic conditions developed over the last decade. Therefore, most studies (82.7%, 24 studies) were published from 2010-2020.

Human derived periodontal ligament (HDPL)stem cells (86%) were predominantly used in the articles included in this review. Periodontitis disturbs the chewing mechanism between teeth and is highly prevalent worldwide. Research on periodontal regeneration was pioneered in 2004, and other stem cells have been reported to have the capability to differentiate into periodontal tissue under proper conditions for induction.^39^

All included studies were conducted in vitro, but 2 studies also included in vivo experiments.^10,35^ Stem cell growth and function are used to determine the optimal in vivo microenvironment conditions, including the oxygen level. Natural cellular microenvironments seem to contain lower oxygen levels, with a mean oxygen level of approximately 12% in arterial blood and 3% in the tissues as well as considerable variation depending on the location. During early pregnancy, the surface of the uterine cavity has approximately 2% oxygen. After utero-placental circulation develops, the placental oxygen level increases to approximately 8%.^1,40^

No uniformity was found between the studies regarding the optimum oxygen concentration/hypoxic conditions for ligament/tendon cultures, but 11 studies (37%) used 2% oxygen and 9 studies (31%) used 1% oxygen. Zhang et al^6^ compared the effects of oxygen concentrations of 1%, 2%, 5%, and 21% and concluded that there was a significant increase in cell proliferation in the severe hypoxia group (1% oxygen concentration) at 24 hours post-cultivation (*P* < 0.05); moreover, after 48 h, cell proliferation was markedly restrained (*P* < 0.05). The level of alkaline phosphatase activity in the severe hypoxia group was also greatly reduced (*P* < 0.05) after 24 hours.

Lastly, this review aimed to evaluate the main outcomes of ligament/tendon cultures under hypoxic conditions. The examined studies did not analyse similar parameters or outcomes; thus, a meta-analysis of the data was not feasible.

Seven included studies (24%) reported increases in cell proliferation.^6,14,16,23,30,35,36^ This result is consistent with that of many other studies that observed a significant increase in the proliferation of embryonic stem cells or adult stem cells exposed to low oxygen tension.^1^

Eight studies (27.5%) reported an increase in the expression of cytokines, such as IL-1β,^20,31,37^ IL-6,^11,20,31,37^ IL-8,^31^ IL-10,^10^ IL-37,^10^ and TNF-α.^37^ However, other studies examined different cytokines. Ito et al. observed no effects on the secretion of IL-1β, IL-6, IL-8, IL-17A, macrophage migration inhibitory factor, monocyte chemoattractant protein-1, TNF-α, and macrophage colony-stimulating factor in the culture media.^22^ The production of the pro-inflammatory cytokine IL-8 was suppressed in MSCs cultured under hypoxic conditions, whereas the levels of the anti-inflammatory cytokines IL-17A and GM-CSF increased following short-term hypoxia.^41^

Other studies identified a significant increase in the production of collagen type I^13^ and III^11,13^ and GAG.^13^ These findings show promise for tissue engineering and offer the option to regenerate the tendon tissue by producing a stronger tendon construct for injury repair, thicker collagen fibre density, denser tissue architecture, and more normal restoration of the tendon-bone interface.

Six studies described a significant increase in VEGF expression.^14,20,24,26,31,34^ When stem cells were placed in low-oxygen conditions, most studies examining MSCs revealed an increase in growth factor secretion, particularly VEGF. Angiogenesis may be induced by stem cells through cell differentiation that contributes to angiogenesis directly or indirectly via hypoxia-stimulated cytokine production.^1,24^

This review has some limitations, including the use of different oxygen concentrations and the predominant use of stem cells derived from the HDPL. Further investigations are needed to determine which oxygen concentrations are effective in achieving the best outcome. Research comparing different oxygen concentrations is needed to identify the optimal conditions for tissue culture.

## Conclusion

Primarily, this systematic review found that a low oxygen concentration is an important factor contributing to the preservation, proliferation, differentiation, and function of ligament/tendon cultures. In a notable study, a significant increase in cell proliferation was observed in the severe hypoxia group (1% oxygen concentration) at 24 hours post-cultivation, but cell proliferation was markedly reduced at 48 h post-cultivation. Areas for future research might include optimising the preconditions in terms of the timing and oxygen concentrations that are most effective for achieving each outcome.

## Ethical Issues

This article does not contain any studies with human participants or animals performed by any of the authors.

## Conflict of Interest

None of the authors have conflicts of interest related to the study to disclose.

## Supplementary Materials

Supplementary file 1 contains Table S1.
