# Supplementary material for: Ligament/Tendon Culture under Hypoxic Conditions: A Systematic Review
Source: Adv Pharm Bull. 2020 Oct 20;11(4):595–600. doi: 10.34172/apb.2021.069 (PMC8642806; doi:10.34172/apb.2021.069)
Supplement: Supplementary file 1 — contains Table S1. [file apb-11-595-s001.pdf]

Table 1. Overview of the studies

No.	Author	Ligament/tendon culture source	Type of study	Method	O2 Concentration	Evaluation	Main outcome measure(s)
1	Matsuda et al[23]	Human periodontal ligament (PDL)	In vitro	Attempting to elucidate the specificity of pathways from environmental stress to cellular outcomes mediated by mitogen-activated protein kinase (MAPK) activation.	5%	Examine the responsiveness of cultured hPDL cells to epidermal growth factor (EGF), hypoxia, and mechanical stress, in terms of cell proliferation, differentiation, and the associated activation of three different MAPKs	Cell proliferation was induced in the presence of 10 ng/mL EGF or hypoxic conditions (5% O_2)<br>Cell proliferation was inhibited by cyclic stretch (9% strain, 6 cycles/min)<br>Alkaline phosphatase activity was increased by cyclic stretch but decreased by EGF and hypoxia.
2	Petersen et al[24]	Achilles tendons from rats	In vitro	Achilles tendons were dissected from rats, primary cultures of rat tenocytes were challenged with different stimuli (hypoxia and PDGF) and VEGF secretion was measured.	5%	Immunohistochemistry, enzyme-linked immunosorbent assay (ELISA)	Stimulation with PDGF increased VEGF secretion 2-fold.<br>Hypoxic conditions alone (5% O_2) increased VEGF secretion only 2-fold.<br>The combination of cytokines and hypoxia increased VEGF production 5-fold.
3	Amemiya et al[14]	Sprague– Dawley rat PDL	In vitro	In the hypoxia group, cells were incubated with 2% O_2 for 1–3 d. In the reoxygenation group, cells were first incubated under the same conditions as the hypoxia group for 24 h and then returned to normoxic conditions and cultured for 1–2 additional days.	20% vs 2%	- Level of expression of the bone sialoprotein and vascular endothelial growth factor mRNAs<br>- Alkaline phosphatase activity	Significantly higher proliferation rates were observed in both the hypoxia and re oxygenation groups than in the control group.<br>Alkaline phosphatase activity was significantly higher in the hypoxia group.<br>The bone sialoprotein mRNA was expressed at significantly higher levels in the hypoxia group.<br>The vascular endothelial growth factor mRNA was expressed at significantly higher levels in the hypoxia group.

Supplementary

4	Motohira et al [20]	Human PDL	In vitro	Human PDL cells were cultured in 3 groups: 1% O_2 (hypoxia), 20% O_2 (normal oxygen tension [normoxia]), or an oxygen concentration that ranged from 1% to 20% (reoxygenation).	20% vs 1% vs Reoxygenation (1% to 20%)	Levels of vascular endothelial growth factor (VEGF), interleukin (IL)-6, IL-1b, tumor necrosis factor-alpha (TNF-α), and prostaglandin E2 (PGE_2) were determined using ELISAs.<br><br>Expression of the corresponding mRNAs was detected using reverse transcription-PCR	Significantly higher extracellular concentrations of VEGF and IL-6 were detected in the hypoxia group.<br><br>The levels of the IL-1b mRNA and protein were only increased in the hypoxia group.<br><br>Neither TNF-α nor PGE_2 was detectable in samples from either group,<br><br>PGE_2, VEGF, IL-6 and IL-1b production was detected after reoxygenation.<br><br>The levels of the secreted VEGF, IL-6 and IL-1b proteins and mRNA also tended to increase after reoxygenation.
5	Kitase et al[33]	Human PDL	In vitro	Gene expression in cultured human PDL cells induced by hypoxia was analyzed	Less than 1%	Gene expression in cultured human PDL cells induced by hypoxia was analyzed using a cDNA array, followed by RT-PCR analysis.	The RT-PCR analysis revealed the upregulation of 6 genes (IGFBP3, CCR2A, CCR2B, MIF, BIGH3 and VEGF) and downregulation of 5 genes (FGF7, TR, FGF2, PDGF1 and CCL2) in the PDL cells exposed to hypoxia.<br><br>The expression of the chemokine receptor CCR2 was increased in PDL cells exposed to hypoxia.<br><br>Hypoxic conditions elicit the expression of pro-apoptotic genes.
6	Park et al[32]	Human PDL	In vitro	PDLFs were transferred to a GasPak pouch, where the total oxygen concentration was reduced to less than 1%, to induce hypoxia. PDLFs were incubated for 24 h in the presence of Desferoxamine to induce HIF-1α expression. PDLFs were	Less than 1%	RT-PCR analysis<br><br>Western blot analysis<br><br>The concentration of soluble RANKL was determined using a human RANKL ELISA.	Hypoxia significantly increased the levels of RANKL mRNA and protein, as well as hypoxia inducible factor-1α (HIF-1α) protein in PDLFs.<br><br>Hypoxia enhances osteoclastogenesis by increasing RANKL expression in PDLFs.

Supplementary

				used at passages four to six in the experiments.			
7	Chae et al[31]	Human PDL	In vitro	The compressive force was adjusted by adding metal slices to the cylinder. PDLFs were subjected to 0.5, 1.0, 2.0 or 3.0 g/cm^2 of compressive force for the indicated periods.<br>PDLFs were transferred to a GasPak Pouch, where the total oxygen concentration was reduced to less than 1%, to induce hypoxia.	Less than 1%	RT-PCR analysis<br>ELISA<br>Luciferase reporter assay	A hypoxic treatment for 24 h increased the levels of the IL-1β, IL-6 and IL-8 mRNAs.<br>Mechanical compression and hypoxia significantly increased the expression of IL-1β, IL-6, IL-8, TNF-α and VEGF in PDLFs.
8	Wu et al[26]	Human PDL	In vitro	The effects of different periods of hypoxia (2% O_2) on the osteogenic potential, mineralization and paracrine function of cocultured PDLSCs were investigated.	2%	Osteogenic potential, mineralization and paracrine function of cocultured PDLSCs<br>ERK1/2 and p38 MAPK activity was measured in PDLSCs cultured under hypoxic conditions using western blotting.<br>The involvement of ERK1/2 and p38 MAPK in PDLSCs was investigated.	Increased osteogenic differentiation of co cultured PDLSCs was observed, as evidenced by markedly increased alkaline phosphatase (ALP) activity and PGE_2 levels, vascular endothelial growth factor (VEGF) release, levels of the runt-related transcription factor 2 (Runx2) and Sp7 mRNAs and proteins and mineralized nodule formation.<br>ERK1/2 was phosphorylated in a rapid but transient manner, whereas p38 MAPK was activated in a slow and sustained manner in cells exposed to hypoxia.
9	Millar et al[11]	Human hamstring tendon tissue	In vitro	Fifteen torn supraspinatus tendons and matched intact subscapularis tendons (representing the 'early pathology') as control samples were collected. Human tendon-	21% vs 1%	The effect of hypoxia upon tenocyte biology was measured ex vivo using quantitative real-time PCR, multiplex cytokine assays, apoptotic proteomic profiling, immunohistochemistry and	- Hypoxic tenocytes exhibited increased production of proinflammatory cytokines (*P*<0.001), altered matrix regulation (*P* <0.01) and increased production of collagen type III through a

Supplementary

				derived primary cells were isolated from hamstring tendon tissue obtained during hamstring tendon anterior cruciate ligament reconstruction.		annexin V staining followed by fluorescence-activated cell sorting.	mitogen-activated protein kinase-dependent pathway.<br>- Hypoxia increased the expression of several mediators of apoptosis and thereby promoted tenocyte apoptosis.
10	Zhang et al[6]	Human PDL	In vitro	Cultured HPLFs (fifth passage) were assigned to the slight (5% O_2), middle (2% O_2), and severe hypoxia (1% O_2) groups and the control (21% O_2) group, respectively. At 12, 24, 48 and 72 h, cell proliferation and ALP activities were evaluated	5%, 2% and 1% vs 21%	Cell proliferation and ALP activities	- Cell proliferation was significantly increased in the severe hypoxia group at 24 h post-cultivation (*P*<0.05).<br>- Cell proliferation was markedly reduced at 72 h post-cultivation (*P*<0.05), and the difference was more marked in the severe hypoxia group (*P*<0.05).<br>- ALP activity decreased at each time point as the level of hypoxia increased. No marked difference was observed between the hypoxic and control groups after 12 h.<br>- The ALP activity of the HPLFs in the severe hypoxia group was markedly reduced (P<0.05) after 24 h.
11	Kato et al[27]	Human PDL	In vitro	Functional gap- junction-mediated intercellular communication was investigated in isolated primary hPDL cells.	2%	The fluorescence recovery and expression levels of Cx43 decreased in a time-dependent manner in cells cultured under the hypoxic condition.	Expression levels of Cx43 decreased in a time-dependent manner in cells cultured under the hypoxic condition. Exposure to a GJ inhibitor or hypoxia increased *RANKL* expression, and decreased *OPG* expression.<br>GJIC is responsible for hPDL cells and that its activity is reduced under hypoxia
12	Zhao et al[15]	Human	In vitro	Osteogenic differentiation, molecular characterization, and various behaviors of PDLSCs	20 vs. 2%	qRT-PCR, Western blot, and ELISA. The effect of endothelial cells (ECs) on	Hypoxia promoted the osteogenic differentiation of PDLSCs and increased EC migration.

Supplementary

		PDL		and human umbilical venous ECs under hypoxia were assessed		The osteogenic differentiation of PDLSCs was assessed using NS398 (cyclooxygenase 2 blocker) and SU5416 (VEGF receptor inhibitor).	NS398 impaired EC migration and prostaglandin E_2 (PGE2)/VEGF release.<br>NS398 (pretreated ECs) decreased PGE2/VEGF concentrations.
13	Jian et al[37]	Human PDL	In vitro	Hypoxia induced TNF-α, IL-1β, and IL-6 expression in human periodontal ligament (hPDL) cells.	1%	Real-time PCR analysis of mRNA expression<br>Inflammatory cytokine levels were measured using ELISAs	Hypoxia increased Pg LPS-induced TNF-α, IL-1β, and IL-6 expression in hPDL cells.<br>The hypoxic environment may enhance the immune function of hPDL cells induced by Pg LPS.
14	Zhang et al[35]	Human PDL	In vitro	HPDLSCs used in these experiments underwent 3 passages. Next, HPDLSCs (passage 3) were exposed to normal oxygen (21% O_2) or hypoxic (2% O_2) conditions and cell proliferation was evaluated.<br>HPDLSCs (passage 1 or 2) were cultured in osteogenic or adipogenic medium.	2% vs 21%	Osteoblastic differentiation was evaluated using semiquantitative RT- PCR of 3 osteoblastic markers: core-binding factor a 1/runt-related transcription factor 2, osteocalcin, and osteopontin.	- The proliferation rate was increased, and an increase in the osteogenic differentiation potential was increased, compared to control cells.<br>- Twelve weeks of transplantation, hypoxia-treated HPDLSCs differentiated into osteoblast-like cells that formed bone-like structures.
			In vivo	After 7 d of exposure to hypoxic and normal conditions, each cell group was transplanted subcutaneously into the back of immunocompromised mice to determine differences in osteogenic function in vivo.	2%	Hematoxylin and eosin staining was conducted.	The mean optical density of newly formed bone in animals injected with hypoxia-treated HPDLSCs was significantly higher than the control group ($P < 0.05$).

Supplementary

15	Gölz et al[12]	Human PDL	In vitro	Human primary PDLs were stimulated with lipopolysaccharide from *Porphyromonas gingivalis* (LPS-PG), a periodontal pathogenic bacterium, under normoxic and hypoxic conditions.	21% vs 1%	Quantitative PCR, immunoblots, immunostaining, and a specific ROS assay were performed to determine the levels of NOX4, ROS, and several redox systems.<br>NOX4 and redox systems were evaluated using immunohistochemistry.	Significantly increased NOX4 levels were observed in PDL cells after hypoxic or inflammatory stimulation.<br>Significant upregulation of ROS and catalase.<br>The interaction of NOX4 and redox systems is crucial for ROS formation, which plays a pivotal role in oral diseases.
16	Zhou et al[16]	Human PDL	In vitro	PDLCs and DPCs were cultured under either normoxic (20% O_2) or hypoxic (2% O_2) conditions.	20% vs 2%	Cell viability assays were performed and the expression of pluripotency markers was detected using qRT-PCR and Western blotting.<br>Mineralization, glycosaminoglycan (GAG) deposition, and lipid droplet formation were assessed using Alizarin red S, Safranin O, and Oil red O staining, respectively.	Hypoxia did not exert negative effects on the proliferation of PDLCs and DPCs.<br>Hypoxia plays an important role in maintaining the stemness and differentiation capacity of PDLCs and DPCs.
17	Li et al[17]	Human PDL	In vitro	hPDLCs were cultured in the presence of 2% O_2 (hypoxia) or 20% O_2 (normoxia) and then subjected to a cyclic in-plane tensile deformation of 10% at 0.5 Hz.	20% vs 2%	1) Cell proliferation was analyzed using flow cytometry.<br>2) Cell ultrastructure was analyzed using transmission electron microscopy.<br>3) The expression of hypoxia-inducible factor-1α (HIF-1α) and osteogenic-related factor runt-related transcription factor 2 [RUNX2], and transcription	Significant increases in HIF-1α, SPP1, RUNX2, and SP7 expression occurred in cells cultured in the presence of hypoxia for 24 h.<br>The MAPK inhibitor (PD 98,059) significantly attenuated hypoxia and cyclic tensile stress (CTS)-induced increases in phospho-ERK1/2 (extracellular regulated kinase 1/2), phospho-JNK (c-jun N-terminal kinase), and phospho-P38 levels.<br>Hypoxia regulates CTS-responsive changes in the proliferation and osteogenic differentiation of

Supplementary

						factor Sp7 [SP7] were analyzed using RT-PCR and Western blotting.<br><br>4) The involvement of mitogen-activated protein kinase (MAPK) signaling pathways was investigated by treating cells with specific inhibitors and performing Western blotting.	hPDLCs by modulating MAPK pathways. Hypoxia-treated hPDLCs may represent an in vitro model to explore the molecular mechanisms of periodontitis.
18	Li et al[29]	Human PDL	In vitro	A periodontal ligament tissue model (PDLtm) was established by establishing 3-D cultures human PDLCs on a thin sheet of a poly lactic-co-glycolic acid scaffold. The PDLtm was treated with hypoxia and/or compression for 6, 24, or 72 h. The conditioned media were applied to cocultures of osteoblast and osteoclast (OC) precursors.	2%	RT-PCR was performed to analyze gene expression.<br><br>Tartrate-resistant acid phosphatase staining was performed to examine OC formation.	Hypoxia significantly upregulated the expression of pro-osteoclastogenic cytokine genes in the PDLtm and increased osteoclastogenesis in the cocultures.<br><br>The combination of compression and hypoxia exerted significantly stronger effects than either stimulus alone.
19	Kowalski et al[13]	Human anterior cruciate ligament	In vitro	Three novel anterior cruciate ligament (ACL)-derived cell populations with the potential for ligament regeneration were characterized. The proliferative and differentiation potential of ligament-forming fibroblasts, collagen gene expression and metabolism in cells cultured under both normoxic and hypoxic environments, and their trophic potential in vitro were examined.	20% vs 1%	Histology and immunohistochemistry<br><br>Assessment of the potential for adipogenic, chondrogenic and osteogenic differentiation<br><br>Gene expression analysis	Hypoxia induced significantly higher expression of both collagen I and III.<br><br>Significantly increased GAG concentrations were detected in hypoxic cultures.

Supplementary

20	He et al[36]	Human PDL	In vitro	PDLSCs were separated and purified using the limiting dilution method and identified using flow cytometry. PDLSCs were cultured under hypoxic or normoxic conditions to observe their cloning efficiency.	20% vs 5%	PDLSC proliferation after exposure to different oxygen concentrations was evaluated using the MTT assay.<br><br>Levels of proteins involved in the p38/MAPK and MAPK/ERK signaling pathways were detected using Western blotting.<br><br>Inhibitors of p38/MAPK or ERK were applied to PDLSCs to observe their effects on clone formation and proliferation.	The proliferation of PDLSCs cultured under hypoxic conditions was higher than the control group (P < 0.001).<br><br>A marked increase in p38 and ERK1/2 phosphorylation was observed in hypoxic PDLSCs compared to the control group (P < 0.05).<br><br>Hypoxia increased PDLSC clone formation and proliferation by activating the p38/MAPK and ERK/MAPK signaling pathways.
21	Chen et al[18]	Human PDL	In vitro	Cells passaged to P4 were used in the experiments.<br><br>The UCSC Genome Bioinformatics database was used to locate the HIF-1α, HIF1A-AS1 and HIF1A-AS2 Genes and to obtain the sequences of the HIF-1α, HIF1A-AS1 and HIF1A-AS2 mRNAs.	20% vs 2%	Western blot analysis<br><br>ALP activity<br><br>RT-qPCR	Expression levels of HIF1A-AS1, HIF1A-AS2, HIF-1α, and osteogenic biomarkers were increased in a time-dependent manner after exposure to hypoxia.<br><br>HIF-1α exerted positive regulatory effects on HIF1A-AS1 and HIF1A-AS2.<br><br>HIF-1α promoted the osteogenic differentiation of PDLCs, and HIF1A-AS2 exerted a negative effect on the osteogenic differentiation of PDLCs.
22	Giacoppo et al[10]	Human PDL	In vivo	The CM was collected from hPDLCs cultured under hypoxic conditions (H-hPDLSC-CM).<br><br>Mice were anesthetized after EAE was induced in mice using myelin oligodendrocyte glycoprotein peptide (MOG).	20% vs 3%	- Immunofluorescence staining of mouse spinal cord tissues<br>- Immunohistochemistry of mouse spinal cord tissues<br>- Western blot analysis of mouse spinal cord tissues	A marked increase in the expression of IL-37 was observed in H-hPDLSC-CM-treated mice. In contrast, IL-17 and IFN-g expression was reduced after treatment with H-hPDLSC-CM<br><br>Oxidative stress, autophagy, and apoptosis markers were detected in mice with EAE after hPDLSC-CM administration.

Supplementary

				Mice were randomly assigned to the following groups: 1) naive group (n = 5): mice did not receive an injection of $MOG_{35–55}$ or other drugs and were only used as controls; 2) EAE group (n = 15): mice were subjected to EAE with no other treatment; 3) EAE + H-hPDLSC-CM (n = 10): EAE mice were intravenously injected (tail) with H-hPDLSC-CM (1.0 mg/mouse) 14 d after EAE induction.		-<br>-	
			In vitro	Murine-origin NSC-34 motoneuron cells mixed with 10% fetal bovine serum were cultured. Cells with a passage number of 30 were used in the present study.<br>Cells were subsequently subjected to injury by creating manual scratches with a 1-ml pipette tip. After injury, the medium was removed, and cells were incubated with fresh serum-free medium for 24 h. Neuronal cells without scratch injury were also included as a control.	3%	Immunocytochemistry of NSC-34 neuron cells<br>Western blot analysis of NSC-34 neuron extracts	Negative staining was observed for TNF-α, COX-2, and iNOS in injured neurons treated with H-hPDLSC- CM.<br>A significant increase in the staining for the anti-inflammatory cytokine IL- 10 was observed in H-hPDLSC-CM-treated injured neurons<br>H-hPDLSC-CM completely suppressed the activation of apoptosis.<br>Western blot data reveal the presence of NT3, IL-10, and TGF-β in cells treated with H-hPDLSC-CM, suggesting a neuroprotective effect of H-hPDLSC-CM on injured neurons

23	Murabayashi et al[19]	Human PDL	In vitro	The properties of periodontal ligament-derived MSCs (PDLSCs) cultivated in serum-free and serum-containing media under hypoxic and normoxic conditions were characterized.	21% vs 3%	Cell growth, gene and protein expression, cytodifferentiation potential, genomic stability, cytotoxic response, and hard tissue generation by PDLSCs in vivo were examined.	Hypoxia did not alter the growth of PDLSCs under serum-free conditions but inhibited their osteogenic and adipogenic differentiation.<br><br>PDLSCs cultured in serum-free culture media were more susceptible to damage following exposure to extrinsic cytotoxic stimuli than cells cultured in medium supplemented with serum.
24	Xiao et al[21]	Human PDL	In vitro	hPDLCs (4th passage) cultured using the tissue culture method were randomly assigned to slight (5% O_2), severe hypoxia (1% O_2), and control (21% O_2) groups.	1% and 5% vs 21%	The optical density values were detected, and the growth curve was constructed.<br><br>A wound healing assay was performed to observe the migration of hPDLCs.<br><br>RT-qPCR was conducted to detect the expression of cementum-related genes and Wnt signaling pathway-related genes.<br><br>RT-qPCR, Western blot, and immunofluorescence staining were performed to detect HIF levels.	The growth rate of hPDLCs decreased as the O2 content decreased, and the morphology of hPDLCs changed in the presence of different O2 concentrations.<br><br>hPDLCs migrate faster in 21% and 5% O2 than in 1% O2.<br><br>The expression of cementum-related genes and Wnt signaling pathway-related genes increased in cells cultured under hypoxic conditions.<br><br>The reduction in the O2 concentration increased the levels of the HIF messenger RNA and protein, and HIF was gradually transported from the cytoplasm into the nucleus in cells cultured with a 1% O2 concentration.
25	Ito et al[22]	Human PDL	In vitro	PDLC cultures were collected. For mRNA experiments and Western blotting, the cells were seeded onto six-well dishes at	Less than 0,1%	Quantitative reverse transcription PCR	PDL cells cultured under hypoxic conditions showed an increase in the expression of C/EBPβ and RANKL messenger RNAs (mRNAs).

Supplementary

				5.0 × 10^5 cells/well, and for small interfering RNA (siRNA) transfection experiments, the cells were seeded in 24-well dishes at 1.0 × 10^5 cells/well.		Expression of RANKL and HIf1<br><br>Secretion of cytokines from the PDL cells<br><br>Expression of RANKL and HIF-1α proteins	Hypoxia did not alter the secretion of IL-1β, IL-6, IL-8, IL-17A, tumor necrosis factor-alpha, macrophage migration inhibitory factor, monocyte chemoattractant protein-1, and macrophage colony-stimulating factor in the culture media.
26	Kifune et al[34]	Human PDL<br>Human embryonic fibroblasts (hEMBF)	In vitro	hPDLF (immortalized fibroblasts derived from human deciduous teeth) and hEMBF (immortalized fibroblasts obtained from a human embryo) were used in this study.	Less than 0.1%	The expression of the ANG, VEGF, stromal cell-derived factor-1 (SDF-1), and HIF-1α mRNAs was measured with a real-time RT-qPCR assay.<br><br>Western blotting<br><br>Cytokine array analysis	Levels of the ANG and VEGF mRNAs were significantly increased in PDL fibroblasts cultured under hypoxic conditions, but not in embryonic fibroblasts.<br><br>Hypoxia increased the productions of ANG and VEGF proteins in PDL fibroblasts.<br><br>The expression of the HIF-1α mRNA was not affected by hypoxia in either fibroblast line, although the level of the HIF-1α protein was increased after exposure to hypoxia.<br><br>Under hypoxic conditions, HIF-1α upregulates synthesis of ANG and VEGF in PDL fibroblasts and promotes angiogenesis.
27	Liu et al[25]	Human PDL	In vitro	Hypoxia was induced by exposing cells to a tri-gas incubator with 3% O_2, cobalt chloride (CoCl2) was used to induce the stabilization of HIF-1α protein.	3%	qRT-PCR and Western blotting.	After treating PDLSCs with hypoxia (3% O2) over different time periods (0, 12, 24 and 48 h), qPCR results revealed that the transcription of *TGF"1* declined after 24 h and continued to decline after 48 h.
28	Yan et al[28]	Human PDL	In vitro	hPDLCs were extracted, cultured, and used between passages 3 and 6.	2%	- Real time PCR and Western blotting were performed to detect relative mRNA and protein levels.	Cells cultured in the presence of 2% O2 exhibited decreased A20 expression and an increased RANKL/OPG (R/O) ratio.

Supplementary

				hPDLCs were transfected with lentivirus A20 for overexpression or silencing studies. Twenty-four hours after transfection, 2.5 µg/mL puromycin was added and the cells were cultured for 24 h to select for positive cells.		- The formation of autophagosomes was measured using TEM.<br>- Osteoclastic differentiation was assessed using TRAP staining and the hydroxyapatite resorption assay.<br>The interactions between different proteins were observed using co-IP.	Autophagy in hPDLCs and osteoclast differentiation and hydroxyapatite resorption areas in mouse bone marrow mononuclear cells (BMMCs) were inhibited by A20. Moreover, A20 inhibited polyubiquitination at K63 and increased polyubiquitination at K48 of TRAF6 to suppress autophagy under hypoxic conditions.
29	Mao et al[30]	Human PDL	In vitro	Hypoxia shifted cell metabolism from oxidative phosphorylation to glycolysis with an accumulation of succinate in the cytosol and its release into culture supernatants.	1%	ALP and Alizarin Red S Staining.	The succinate supplement increased hPDLC proliferation, migration, and osteogenesis, but decreased succinate dehydrogenase (SDH) expression.<br><br>The addition of the succinate supplement to cell cultures promoted intracellular succinate accumulation while stabilizing hypoxia inducible factor-1α (HIF-1α), leading to a state of pseudohypoxia.<br><br>The succinate supplement altered cell metabolism in hPDLCs, induced a pseudohypoxia condition, and increased the proliferation, migration, and osteogenesis of mesenchymal stem cells in vitro.

CM, Conditioned medium

Abbreviations:

1.

a. CM — Conditioned medium
b. DMEM — Dulbecco's Modified Eagle's Medium, HG-DMEM: High-glucose DMEM
c. EV — Extracellular vesicles
d. FBS — Fetal Bovine Serum
e. LPS — lipopolysaccharides
f. MCP — Monocyte Chemotactic Protein
g. RANKL — Receptor Activator of Nuclear Factor-kappa B
h. A20 — TNF-α-induced protein 3 (TNFAIP3)
i. TRAF — Tumor Necrosis Factor Receptor

Supplementary

j. EC Endothelial Cells
k. NS398 Cyclooxygenase 2 blocker
l. SU5416 Vascular Endothelial Growth Factor receptor inhibitor
m. VEGF Vascular Endothelial Growth Factor
n. PD98059 Extracellular Signal-regulated Protein Kinase [ERK] inhibitor
o. PGE_2 Prostaglandin E_2
p. BCK-2 B Cell Lymphoma 2
q. TEM Transmission Electron Microscopy
r. TRAP Tartrate-resistant Acid Phosphatase
s. Co-IP Coimmunoprecipitation
t. CCL2 CC chemokine ligand 2
u. IGFBP3 Insulin-like Growth Factor Binding Protein 3
v. MIF Macrophage Migration Inhibitory Factor
w. BIGH3 TGFβ in human clone 3
x. TR Thyroid Hormone Receptor
y. EAE Experimental Autoimmune Encephalomyelitis
z. *COX* *Cyclooxygenase*
aa. *iNOS* inducible Nitric Oxide Synthase
bb. GJIC Gap-junctional intercellular communication
cc. ANG Angiogenin
dd. *NOX4* NADPH Oxidase 4
ee. NAC N-acetylcysteine
ff. HIF-1α Hypoxia-inducible Factor-1α (HIF-1α)
gg. TGF-β Transforming Growth Factor-β1
hh. ELISA Enzyme-linked immunosorbent assay
ii. PCR Polymerase Chain Reaction, RT-qPCR: Real-Time Quantitative Polymerase Chain Reaction

2. Related to specific tendon/ligament:

a. ACL Anterior Cruciate Ligament
b. periodontal ligament stem cells PDLSCs
c. IS Infraspinatus
d. SDFT Superficial digital flexor tendon
e. SL Suspensory ligament
f. SS Supraspinatu

Supplementary
